# Arts in Medicine Partnerships: Interdisciplinary Collaborations to Support Behavioral Health

**DOI:** 10.3390/bs15081030

**Published:** 2025-07-29

**Authors:** Gaelynn Patricia Wolf Bordonaro, Julie Galliart, Kate Van Steenhuyse, Haoyu Huang, Ash Tamzin

**Affiliations:** 1 Art Therapy Program, Counselor Education, School of Applied Health Sciences and Communities Healing Through Art (CHART), Emporia State University, Emporia, KS 66801, USA; 2 School of Medicine, University of Kansas, Wichita, KS 67214, USA; jgalliart@kumc.edu; 3 Kansas Arts Commission, Topeka, KS 66612, USA; kate.vansteenhuyse@ks.gov; 4 Wyandot Behavioral Health Network, Kansas City, KS 66112, USA; 5 Cornerstones of Care Kansas, Kansas City, KS 66102, USA

**Keywords:** art therapy, arts in medicine, creative arts therapies, medical art therapy

## Abstract

The Emporia State University (ESU) /Kansas Arts Commission (KAC) Arts in Medicine Partnership exemplifies interdisciplinary collaboration and the capacity of art therapy to impact mental health and well-being. Through the partnership, art therapy services were offered to medical agencies across the state of Kansas. Participants included medical patients, families, caregivers, staff, and professionals. The article introduces (1) the profession of art therapy and the subspecialty of medical art therapy, (2) the ESU/KAC Arts in Medicine Partnership, (3) examples of positive psychology-informed arts-based experiences, and (4) a pilot study designed to explore the impact of group art therapy sessions with medical teaching faculty.

## 1. Introduction

Art therapy is a mental health profession that combines the constructs of psychology with creative expression to help individuals explore and express their inner worlds, reduce stress, improve mental well-being, and address psychological or emotional challenges. Like social workers and counselors, art therapists are master’s-level mental health professionals with extensive graduate-level training (60 credit hours) and at least 700 h of supervised internship experience; registered board-certified art therapists are required to pursue ongoing post-graduate professional development and document continuing education. Facilitated by a credentialed professional, art therapy “enriches the lives of individuals, families, and communities through active artmaking, creative process, applied psychological theory, and human experience within a psychotherapeutic relationship” (AATA, 2024, para. 1). Art therapists facilitate intrapersonal exploration and interpersonal relationships and work with individuals to find meaning in their artwork. Traditional theoretical practices and psychotherapeutic techniques intersect with creative processes to offer individuals opportunities to express themselves in ways that words may not fully capture. In fact, art therapy is particularly helpful for those who find it difficult to communicate verbally (Rubin, 2009). Like other mental health clinicians, art therapists have master’s degrees. As part of their training, graduate art therapy students complete at least 700 h of supervised internship. In the United States, the profession is regulated by the Art Therapy Credentials Board (ATCB), as well as regulatory boards in states with art therapy licensure. Art therapists study the psychological properties of diverse art media and follow specific standards of ethical practice regarding the display, sharing, exhibition, and ownership of participant art, involving informed consent to photograph or document art products, as well as confidentiality.

The arts can play an essential role in identifying and addressing the psychosocial needs of individuals and creating safe spaces. Art therapy is used to treat a wide range of mental health issues, including depression, anxiety, trauma, Post-traumatic Stress Disorder (PTSD), grief, and addiction. Arts-based intervention can be designed to improve cognitive and sensorimotor functions, foster self-esteem and self-awareness, cultivate emotional resilience, promote insight, enhance social skills, reduce and resolve conflicts and distress, and advance societal and ecological change (AATA, 2024).

Art therapists work in many diverse settings in which counselors and psychologists practice. Sites include in- and outpatient psychiatric settings, hospitals and clinics, schools, addiction and substance abuse treatment programs, shelters, community settings, agencies serving veterans, jails and prisons, private practice, and more. Medical art therapy is a rapidly growing subspecialty of art therapy (Malhotra, 2022). Across the globe, art therapists serve patients and their families in addressing the psychosocial impact of illness or injury as well as the emotional toll of symptom management, treatment protocols, uncertainty, and physical and functional body changes.

Medical settings are often associated with traumatic experiences. For patients, trauma experiences may be impacted by isolation, fear, medical procedures, alteration of body image, separation, loss of control, grief, helplessness, hopelessness, financial anxiety, and death. Medical professionals, families, and caretakers may experience secondary trauma, as well as stress specific to their roles and responsibilities. In the absence of protective factors, trauma can disrupt brain development and have lifelong consequences on physical and psychological health (Shonkoff & Garner, 2012). The effects of trauma result in a diminished ability to (1) cope with daily stressors, (2) build interpersonal trust and establish and benefit from relationships, (3) manage cognitive processes, (4) regulate behavior, (5) control the expression of emotions, (6) maintain physical health, and (7) experience well-being (SAMSHA, 2014).

## 2. Medical Art Therapy

Potash (2018) argued that “the United States-European model of art therapy might very well have originated in a medical facility” (p. 58). Potash recounted artist Adrian Hill’s use of personal art-making when he was hospitalized and recovering from tuberculosis. Hill recognized the healing power of art and envisioned art playing an integral role in mind and body health across medical settings. In *Art Verses Illness* (Hill, 1948), Hill documented the birth of the field of art therapy in the United Kingdom. Malchiodi (1993) defined medical art therapy as “the use of art expression and imagery with individuals who are physically ill, experiencing trauma to the body, or who are undergoing aggressive medical treatment such as surgery or chemotherapy” (p. 66). Medical art therapy was initially specific to engagement with patients. Kaiser (2017) noted the diverse contexts of art therapy in medical settings and its applications with individuals of all ages and medical conditions. She argued “art therapy in medical and healthcare settings has grown to become probably the largest area of published research in the field” (p. 53). Researchers advocate for art therapy alongside conventional medical care to promote patient well-being (Jalambadani et al., 2025; Azmawati et al., 2018; Czamanski-Cohen et al., 2019). Contemporary patient-centered care models have embraced care of the whole person as well as attention to the well-being of all members of treatment teams, including families, caregivers, patients, medical professionals, and agency staff. As a result, medical art therapists work across the continuum of care.

## 3. A Medical Art Therapy Model in Kansas: The Arts in Medicine Partnership

Although some medical centers and hospitals employ full-time site-specific art therapists, other models share resources. As an example, the art therapy program at Emporia State University (ESU) partnered with the Kansas Arts Commission (KAC) to create the Arts in Medicine program through a specific category of KAC funding established to support cross-disciplinary arts-based programs designed to improve Kansas communities and citizens’ lives. KAC, in partnership with the National Endowment for the Arts (NEA), offers “a range of programs and services to support cultural programming across Kansas and enhance the role the arts play in all levels of education, community service, workforce development and quality of life” (Kansas Arts Commission, 2025, para. 2). In Kansas, the arts agency is a part of the Kansas Department of Commerce; readers in other states may seek partnerships with parallel arts agencies. The ESU/KAC Arts in Medicine Partnership serves as a model that could be initiated in other states to serve rural communities or collectives of small agencies.

The ESU/KAC Arts in Medicine partnership exemplifies interdisciplinary collaboration and the capacity of the arts to impact well-being. Now in its sixth year, the Arts in Medicine partnership makes art therapy programming available to medical agencies and institutions in Kansas. Agencies can apply to receive (1) group art therapy programming for patients, caregivers, and medical personnel, (2) individual art therapy sessions for patients receiving medical treatment, (3) arts-based self-care and resilience-building workshops for medical professionals and clinicians-in-training, (4) advocacy and awareness-building collaborations to increase the understanding of the role of art therapy in medical settings, and (5) workshops on the ethical mandate of self-care for medical, mental health, and public service professionals. This unique collaboration has brought highly valued services to patients, caregivers, and medical professionals across Kansas.

Through the Arts in Medicine partnership, medical agencies apply for art therapy services through an online portal. The application process includes questions about the population(s) served and the objectives of programming sought. Each year, art therapy services are facilitated primarily by two second-year ESU graduate art therapy students who have been engaged as graduate assistants (GAs) and receive a stipend and tuition waivers. The partnership funds the GA stipends and tuition waivers as well as art supplies for the programming. Some programming is weekly, but many applicants seek monthly sessions or workshops. The GAs carefully puzzle together schedules that group services by location and agency needs to fit in their 20 h per week positions. In addition to direct services, the scholar interns serve as collaborators and integral members of research protocols and meet regularly for supervision with their professor. The art therapy professor also provides direct service as needed, primarily for single self-care and well-being workshops for teachers, nurses, and other community-based professionals.

Initially, the programming was supported by matching funds from ESU and KAC. Internal support from ESU’s was granted through the Office of Research and Grants via Research and Creativity grants. KAC funding was supported by the National Endowment for the Arts (NEA). After years of successful engagement, NEA policy changed, and matching funds were no longer required; ESU shifted to covering tuition waivers only.

### Positive Psychology, Art Therapy, and the Arts in Medicine Partnership

The Arts in Medicine programming was grounded in positive psychology theory and its intersection with art therapy. The diversity of populations served, brief and sometimes single-session formats, and the potential for dual relationships among participant groups members meant that some theoretical approaches would present ethical problems. For example, groups of co-workers participated together, and patients and medical staff engaged in art-making and processing together; it would be inappropriate to engage in deeply personal approaches or to explore personal trauma or intimate relationships. Additionally, for arts-based well-being and thematic workshops, participants worked together for a single time. The approach needed to be strengths-based and highlight habits of self-care.

Developed (Seligman, 2002) and refined (Seligman, 2011) by Martin Seligman, positive psychology focuses on the study and promotion of well-being and optimal functioning. The approach emphasizes individual strengths, virtues, and positive experiences that contribute to a fulfilling life. The goal is to explore and enhance life experiences that result in a sense of purpose, satisfaction, and positive relationships. Its practice requires “therapeutic essentials such as warmth, accurate empathy, basic trust, genuineness, and rapport” (Seligman, 2011, p. 40). Seligman (2011) identified five distinct and measurable elements that, underpinned by signature strengths and virtues, contribute to a fulfilled life. These elements are collectively known as the PERMA model, which stands for positive emotions, engagement, positive relationships, meaning, and accomplishments.

Isis (2016) argued that art therapy naturally complements positive psychology. She highlighted art therapy interventions that underscore the elements of the PERMA model. She wrote that “the integration of art therapy with positive psychology is a rich and valuable collaboration toward the pursuit of well-being and optimal functioning…art therapy has the potential of motivating clients to reveal more of who they are to themselves” (p. 97).

Informed by positive psychology, Arts in Medicine interventions were designed to engage participants in (1) reflecting upon personal skills, strengths, gifts, and talents; (2) recalling the values and passions they treasured; (3) creating habits of gratitude and reflecting on experiences that bring joy, comfort, or contentment; (4) experiencing and nourishing opportunities for self-care; (5) nourishing personal connections and building community; and (6) finding meaning through art-making and reflection.

## 4. ESU/KAC Arts in Medicine Programming: Examples of Medical Agencies and Arts-Based Positive Psychology Interventions

Applications for art therapy programming have been submitted primarily by medical agencies in east central Kansas. Examples include medical hospitals, children’s hospitals, cancer-care centers, community-based cancer support groups, a university medical school, clinics that provide medical and behavioral health services, HIV clinics, nursing homes, and rehabilitation facilities. Developmental day programs have also applied, and when possible, received services. Below are three examples of settings with brief glimpses into the role of the arts and art therapy in medicine.

*Hospital-based Programming:* Each year, at least one hospital has requested weekly individual art therapy visits for patients rehabilitating post-surgery. In hospitals and with any population that may be immuno-compromised, it is essential to provide only art materials that have been sterilized; individual sets of art supplies can be ideal so patients do not share materials. Pastels and media that can be inadvertently inhaled are strictly avoided. A favorite art experience that is manageable by patients restricted to their beds has been handmade cards. The art therapists bring supplies including kits with washi tape, stickers, craft paper, glue sticks, and blank note cards with envelopes. The supplies do not risk spills or messes, are non-threatening and have high success, and can engage individuals at the level they feel comfortable. Patients who craft as a hobby can jump right in and make meticulously constructed, carefully assembled cards. Non-artists enjoy the simplicity and esthetic of the washi tape and stickers. The art therapists can help take dictation if the patient prefers, and stamps are available to mail the cards, too. Patients have thoroughly enjoyed (1) the non-medical one-on-one visits of the art therapists-in-training, (2) connecting with family and friends from whom they have been isolated, (3) the escape from stress afforded by the simple arts-based experience, (4) connecting with the art therapist-in-training as they worked, (5) the opportunity to make a thank you card (their suggestion) for nurses and caretakers who have made their hospital experience pleasant, and (6) the human connection they experience in what can be a sterile and lonely setting.

Occasionally, as they work on cards, patients share their stories and directly process their medical experiences. The art therapists-in-training are prepared to listen, witness, engage, support, and refer if appropriate (since their role at the hospital is limited to just a couple hours each week). Their graduate-level mental health training prepares them to engage and respond appropriately to expressions of fear, pain, angst, isolation, frustration, anger, and other emotions clients may communicate verbally or through non-verbal symbol systems in art.

Importantly, simple media and craft practices are utilized purposefully in art therapy to start where the participant is. Art therapists have used a wide range of craft experiences in their practice. Craft practices in art therapy can play an important role in recognizing individuals’ interests and identities (Anderson, 2021; Woolhiser Stallings & Clark, 2021), ameliorating concerns about talent or creative capacity, practicing self-care (Yi, 2021), addressing systemic oppression (Holmes, 2021; Mageary, 2021), and building therapeutic rapport and relationships (Kapitan, 2021). Leone (2021) argued that craft in art therapy “can serve as a powerful and transformational tool…and play[s] a valuable role in socially just art therapy practice” (p. xxiv).

*Community-based Self-care Workshops*: An example of a requested self-care workshop was one designed for 911 dispatchers; the workshop was part of a comprehensive plan by their agency to support dispatcher well-being. The daily work of the dispatchers involved regular exposure to life-threatening emergencies, distressed callers, and traumatic situations. Systemic mental health strategies are essential to avoid burnout, anxiety, and even PTSD; furthermore, the mental health and well-being of dispatchers affects the core of public safety operations (CentralSquare, 2024). In fact, mental health support is crucial in “maintaining the effectiveness of emergency response services” (para. 3). Mental acuity and emotional stability directly impact dispatchers’ capacity to process information rapidly and accurately.

**Figure 1:**
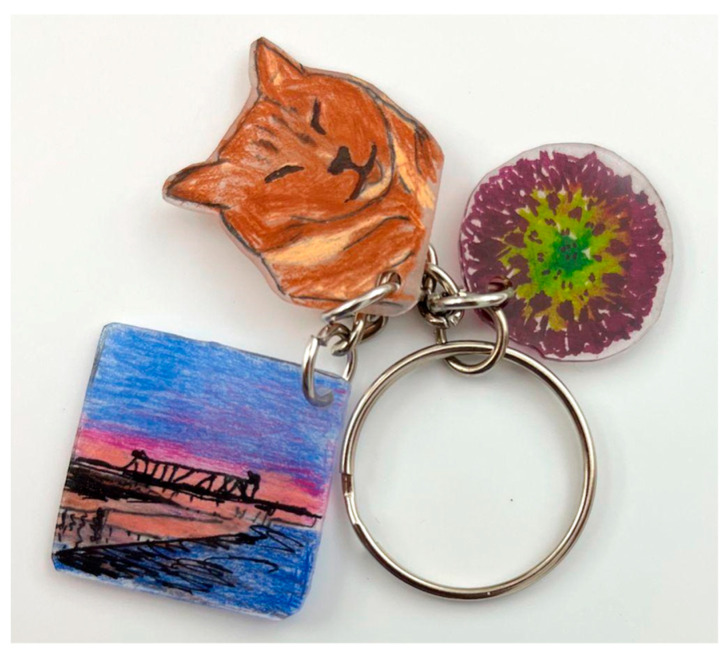
Shrinky Dinks^®^ keychain, example.

**Figure 2:**
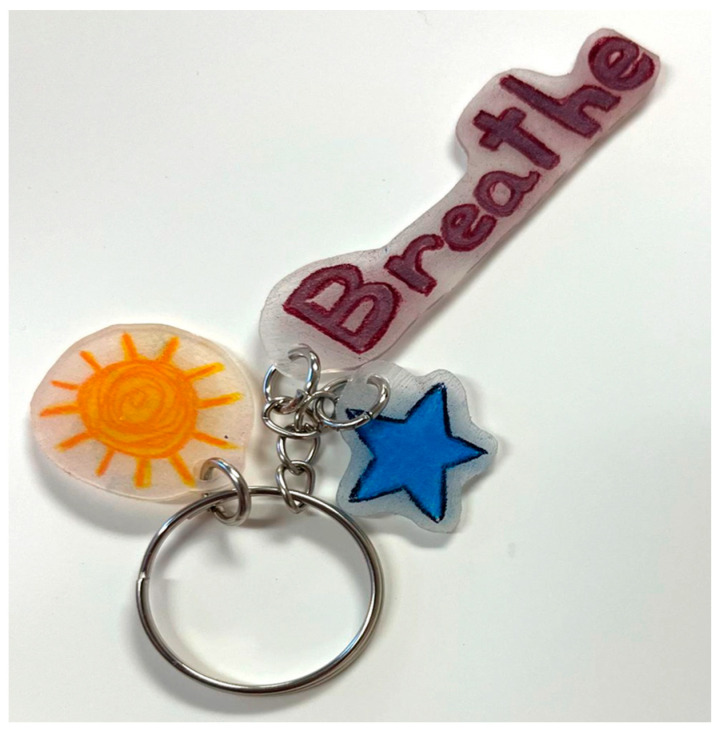
A second example of a Shrinky Dinks^®^ keychain.

A novel art media, Shrinky Dinks^®^, was introduced in the workshop for the dispatchers; the psychoeducational workshops (1) introduced and reinforced self-care not as luxury but as an ethical mandate (Wolf Bordonaro, 2023) and (2) highlighted the impact of stress and trauma as well as intra- and interpersonal signs of stress. Novelty can be an essential part of internalizing psychosocial and psychoeducational constructs, Shrinky Dinks^®^ can serve as an ideal therapeutic media for both individuals familiar with arts practices and individuals who do not identify as artists. Participants draw an image on thin sheets of plastic which shrink to about a quarter to a third of their size and harden when heated to 325 °F. In mental health work, the pieces made with the novel media are wonderful metaphors for shrinking problems into a manageable size, they serve as tangible reminders of important concepts, and they function as meaningful and personal symbols of growth, change, and identity (Lamb, 2018; Wolf Bordonaro et al., 2009). The dispatchers in the workshops were invited to create two symbols they could carry with them as key chains, zipper pulls, or phone charms (see Figure 1 and Figure 2). The first was a symbol reflecting the values, gifts, talents, and/or skills they brought to their profession; the second was a symbol for something they did for self-care or that brought them joy. When they were finished, the participants shared their symbols and engaged in dialog about why they entered the field and why they stayed. They also shared stories about how they relaxed and recentered when not at work and steps they could take to commit to a practice of self-care.

**Figure 3:**
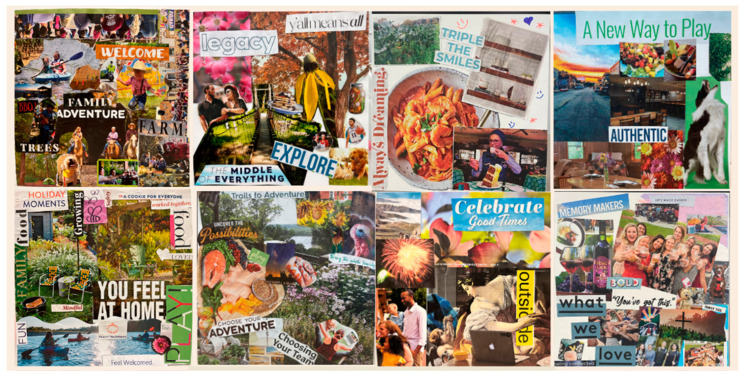
Examples of supportive community collages.

**Figure 4:**
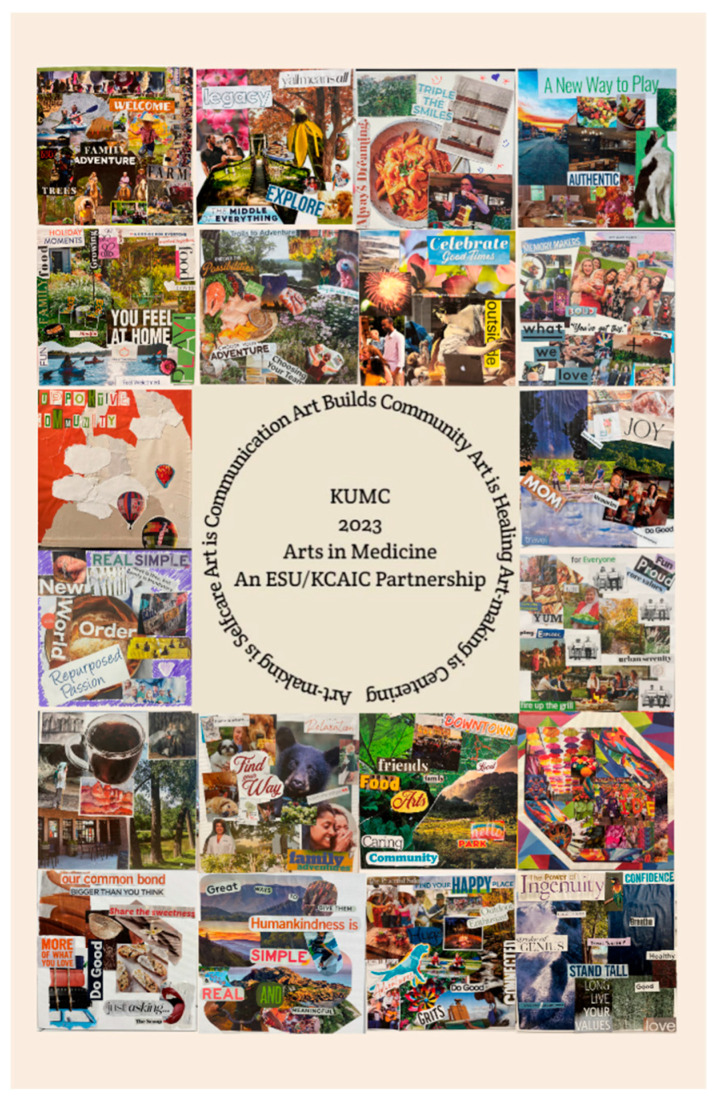
A poster made with a collection of participants’ supportive community collages.

*Art Therapy Programming with Hospital Medical Staff*: During multiple years of the partnership, hospitals have applied to receive arts-based resiliency and burnout prevention experiences for medical providers and staff. Art therapy interns established regularly scheduled therapeutic art-making sessions and pop-up studios (Inman, 2018), as appropriate for the hospitals and the target participants. The principles of positive psychology were directly applied to the planned arts-based interventions. A sense of community and belonging is a fundamental concept in positive psychology; feelings of connectedness contribute to overall well-being. Reinforcing social and community connection and engagement through art offers opportunities to experience a sense of purpose, recognize shared values and goals, and practice civic responsibility and solidarity. Groups of participating medical professionals, including doctors, nurses, and administrators, have created supportive community collages. Participants were invited to choose images, symbols, words, and colors from magazines to represent their perceptions of a supportive community. In their art-making, participants drew upon personal experiences of connection to the community, as well as personal concepts that represented supportive communities. Each participant worked independently on their own 8″ × 8″ collage. When they were finished, they gave permission for their collages to be photographed and shared with one another, and each kept their own piece (see examples in Figure 3). Rich dialog ensued as physicians recognized their personal values and needs in others’ artwork. They discussed plans to act upon the themes in the artwork, including nurturing supportive family and work relationships, the active use of parks and other natural resources in the community, and embracing opportunities to pursue meaningful downtime. They expressed new appreciation for the intersections of their professional, social, and broader communities with their own sense of well-being. The art therapy facilitators used the photographs to create a poster with all of the images (see Figure 4). Copies of the poster were printed on heavy 11″ × 17″ paper and distributed to each of the participants. The response to the final collaborative piece was inspiring. The poster, which many reported they would hang in their offices, reinforced plans they made to take action on one of the fundamental principles of positive psychology. This art experience was introduced to a group of medical staff who met as regularly scheduled groups, but it could be implemented as pop-up studio events as well.

### ESU/KAC Arts in Medicine Partnership Pilot Research Initiatives

In 2022, the University of Kansas (KU) School of Medicine, Wichita, applied to receive art therapy services. KUMC is a premier medical training institution in Kansas which strives to instill a spirit of collaboration, curiosity, and positivity. The application requested art therapy services focused on the well-being and resilience of medical faculty comprising physicians and basic scientists. Qualitative interviews with participants in the initial program informed subsequent applications for services, and in 2023, the collaboration led to opportunities to engage in exploratory research.

A pilot study undertaken for quality improvement was designed to explore the impact of group art therapy sessions with medical teaching faculty. Research questions explored the impact of art therapy on participants’ positive emotional states. Participants (N = 57) from two KU medical school campuses were recruited and divided into seven groups; each group met once a month for six months. Some groups were comprised entirely of participants who identified as women, and some were mixed. Other intersectional identities within each group were diverse. Based on qualitative data from year one of the partnership, participants were invited to indicate their preference for participating in a gender-specific or mixed group and were matched accordingly.

At the start of each monthly session, participants were given the option to voluntarily complete the Internationally Reliable Short-Form of the Positive and Negative Affect Schedule (I-PANAS-SF) (Thompson, 2007) to measure changes in affect as a result of participation in the monthly art therapy groups. The I-PANAS-SF inventory lists ten different words that describe feelings and emotions. As a baseline measure and during each art therapy session, participants indicated (on a five-point Likert Scale) the extent to which they had experienced each of the ten emotions in the weeks leading up to each session. Participants were shown a QR code and completed the online survey through their cell phones.

The psychoeducational dialogs and art therapy interventions introduced in the six sessions focused on the principles of positive psychology. Some of the directives were introduced in previous narratives. Importantly, intervention activities were designed by a credentialed art therapist and facilitated by clinician-supervised students pursuing their master’s degrees in art therapy. Art therapy interventions should only be facilitated by trained art therapy clinicians. Table 1 introduces additional media and directives.

Preliminary results were promising, but complications limited opportunities for statistically relevant conclusions. There simply was not enough data collected. A primary challenge was the physicians’ necessarily irregular participation in the art therapy groups. Medical responsibilities and other conflicts (committee meetings, patient rounds, acute or pressing patient needs, etc.) understandably trumped engagement in self-care experiences and follow-up on survey completion. Nonetheless, participants articulated appreciation and enthusiasm for their engagement in the arts-based interventions. They described the art therapy sessions as “inspiring, satisfying, incredibly positive, relaxing, enjoyable, therapeutic, affirming, calming, mindful, rejuvenating, and… absolutely amazing”. Additionally, in exit interviews, 96% of survey participants reported that “if art therapy was offered to faculty again”, they would participate. The same percentage reported that “if art therapy was offered to faculty again, they would recommend the experience to a colleague”. Table 2 lists faculty sentiments in response to the question “What were positive aspects of your experience?” This item was not originally planned as part of the exit interviews. We realized the challenges to participation, data collection, and data analysis invited opportunities to plan ahead for a future study. Participants’ responses informed arts-based intervention planning for subsequent programming and research. Only twelve participants made themselves available to complete both the intake and exit interviews. All twelve offered responses to the item; Table 2 captures their responses.

Opportunities for pilot studies, and perhaps empirical studies in the future, are certainly an added benefit of the partnership. Formal protocols, the use of instruments, and informed consent are reviewed by Institutional Review Boards. Importantly, even when arts-based programming does not include research designs and data collection, informed consent was obtained to photograph participants’ artwork to share in supervision sessions as well as educational forums such as publications and conference proceedings.

## 5. Programming Recommendations and Opportunities to Improve Research Protocol

Each year of the ESU/KAC partnership informs subsequent years. One of the earliest and most important realizations was to articulate the offering of art therapy programming and services not only for patients but for individuals across the continuum of care. With this clarification in the call for applicants, opportunities for medical professionals were highly sought.

An important realization during and in the aftermath of COVID-19 was the need to offer self-care workshops for medical professionals and staff. These offerings expanded to other professionals, including mental health clinicians, emergency dispatchers, and educators. An essential component of the arts-based workshops was the introduction and reinforcement of self-care as an ethical mandate, not a luxury. Medical, mental health, and education professionals all follow Codes of Ethical Conduct, but they rarely prioritize personal well-being. Through the Arts in Medicine partnership, personal art-making and self-care practices were regarded as critical for the ethical provision of medical, educational, and mental health services.

Through our pilot study in partnership with KU School of Medicine, we have improved our design to study change in positive affect. We discovered the value in measuring the change in positive affect in-time at the session level instead of over time at the program level. We originally hoped that participating consistently in a small group over time would show changes in positive affect, but we did not receive enough completed inventories to be able to run the analyses we had planned. Inconsistent attendance further limited data from the already small sample size. For example, out of over 50 participants in the pilot, we only had 12 individuals complete both the first and final inventories. In subsequent partnership years (2024 and 2025), we have been asking participants to complete inventories at the start and end of individual sessions. These inventories are paper versions, distributed to each member with designated time allotted to complete the paper versions at the beginning and end of each session. This method is proving more effective than asking the participants to find time to complete the online inventories after each session. These changes have resulted in increased inventory completion and the ability to link changes in positive affect to participation in art therapy activities. As we move forward, the limits of small sample size will not be compounded by challenges to consistent participation and the completion of pre- and post- surveys. As a result, in the future, we will be able to complete a statistical analysis of the survey responses and results.

## 6. Conclusions

In addition to the positive impact of direct mental health services, the KAC/ESU Arts in Medicine partnership has generated multiple tangible outcomes, including an art exhibit at Wesley Children’s Hospital in Wichita, KS, presentations at conferences of the American Art Therapy Association (Wolf Bordonaro et al., 2019a, 2019b, 2023), the National Association of State Arts Agencies (Jasso, 2018), and the Association of American Medical Colleges Group on Women in Medicine and Science (Galliart & Frazer, 2022), multiple workshops at the MUHAS Teaching Hospital in Dar es Salem, Tanzania, an article in the NASAA Newsletter, two master’s projects (Lamb, 2018; Inman, 2018), and a pilot intervention guide/book. The Arts in Medicine Partnership has also made possible rich internship experiences for the ten graduate art therapy students who have worked closely with faculty to organize, design, and facilitate the medical art therapy programming. Finally, programming has served medical professionals, patients, staff, families, and caretakers with creative mental health interventions designed specifically to address their needs.

The ESU/KAC Arts in Medicine partnership represented an exciting cross-disciplinary collaboration in the provision of mental health services. Agencies who applied for art therapy services worked directly with the ESU Graduate Assistants and faculty to identify agency needs, appropriate programming, and frequency of services. Kansas Arts Commission administrators worked with the art therapists to target programming, solicit applications from agencies and institutions, share the program’s scope of practice with stakeholders, and demonstrate the unique applications of arts professions to state leadership. Research partners from the University of Kansas Medical Center worked with the ESU art therapists and art therapists-in-training to support the well-being of teaching physicians and medical residents, as well as to design and initiate formal studies. Teaching physicians at KUMC sought direct services for their in- and outpatients. Agency activity coordinators engaged with art therapists to learn adaptive programming for geriatric patients and individuals with disabilities. Counselors working in medical agencies worked with the art therapists to plan programming and events. Teams of art therapists, medical personnel, arts administrators, and researchers worked together to present at national conferences, author articles, and offer panel presentations and in-services. The art therapy faculty is working with a KUMC administrator to hire an art therapist to serve KUMC stakeholders (faculty, students, patients, and community partners). Art therapy itself is a cross-disciplinary profession which marries art-making experiences with psychological constructs.

The partnership and resulting collaborations have introduced the field of art therapy to a range of stakeholders across the state of Kansas. Direct engagement of patients and members of the medical community in psychosocial and psychotherapeutic experiences that centered art-making, creative processes, applied psychological theory, and lived experience demonstrated the significant role and meaningful impact of art therapy on mental health and well-being across the continuum of medical practice and care.

## Figures and Tables

**Table 1:** Additional examples of positive psychology-informed art directives using non-traditional art media.

Art Experience	Directive	Materials	Therapeutic Concepts
Pocket Gratitude Journals or Gratitude Tins	Participants were invited to decorate or customize the cover of their gratitude journals or tins. Prompts may include asking them to think about how they define gratitude; memories, ideas, experiences, things, or people that make them feel grateful, and images/symbols that are meaningful. To create a habit of gratitude, individuals make time every day to add entries (words or symbols) that document (large or small) experiences of gratitude.	Stickers, washi tapes, scissors, stamps, drawing materials, pocket size blank books or mini hinged tins, paper	Gratitude, interpersonal connection, mindfulness, positive experiences, “making special” (Dissanayake, 1992)
Flowerpots as Surfaces for Art-Making	Participants were invited to decorate or customize the pot by illustrating (collaging, drawing, painting) essential elements that support growth in their lives. Ask them to think about what they need to grow, change, or transform. Alternatively, create 3D printed pots. A next step could be to plant and nurture seeds.	Flowerpots, paint, paint brushes, markers, collage materials, glue	Metaphor, support, growth, self-care, finding meaning, resilience
Rocks as Surfaces for Art-Making	Rocks are sturdy objects that can “hold” difficult psychological content or can simply serve as a canvas for patterns or dots. Participants were invited to paint mandalas or other patterns on the rock. Ask participants to maintain awareness of feelings and thoughts going through their mind and to focus on the movement of their hands, the emerging of the patterns, and the sounds of the dotting tool on the rock.	Rocks, paint pens, paint, acrylic paint, brushes, dotting tools, pencils	Mindfulness, rhythm, repetition, mandala-making, centering, self-care
Takeaway Tee Shirts	Participants were asked to use fabric markers on provided tee shirts to write (or draw) positive messages, “ah ha” moments, new ideas, or other takeaways from the series of sessions. When the paint from the markers dried, participants tied and dyed their shirt into a desired (or random!) design. Kits of dyes in bottles made the process easier to control and was lessmessy than traditional dipping techniques.	T-shirts, spray or bottle tie-dye, fabric markers, rubber bands, trays or table coverings	Lifelong learning, reflection, sustaining change, self-care, well-being, wearable art as communication

**Table 2:** Responses of participating physicians to the question “What were some of positive aspects of your (art therapy) experience?”

“What Were Positive Aspects of Your (Art Therapy) Experience?”
Art therapy “was a real wellness activity that had an impact on people.”
Art therapy “felt like a break in my week.”
Art therapy was an “opportunity for reflection [and a] chance to think deeply.”
Art therapy provided “camaraderie [and] permission to be silly and creative without judgement.”
Art therapy engendered “feeling a sense of community.”
In art therapy, “I enjoyed having the down time to be creative, reflect, and connect with others.”
Art therapy meant “making time to decompress… [and] feeling valued.”
“Working with the art therapists” was the highlight of the experience.

## Data Availability

No new data were created or analyzed in this study. Data sharing is not applicable to this article.

## References

[B1-behavsci-15-01030] AATA (2024). What is art therapy?.

[B2-behavsci-15-01030] Anderson M., Leone L. (2021). Queer ethos in art therapy. Craft in art therapy: Diverse approaches to the transformative power of craft materials and methods.

[B3-behavsci-15-01030] Azmawati M., Boekhtiar B., Zawiah M., Aisah S., Chiew W., Dalila R. (2018). The efficacy of art therapy on mental health outcomes among breast cancer patients: The current state of evidence based on randomised controlled trials. IIUM Medical Journal Malaysia.

[B4-behavsci-15-01030] CentralSquare (2024). The need for mental health support for 911 dispatch.

[B5-behavsci-15-01030] Czamanski-Cohen J., Wiley J. F., Sela N., Caspi O., Weihs K. (2019). The role of emotional processing in art therapy (REPAT) for breast cancer patients. Journal of Psychosocial Oncology.

[B6-behavsci-15-01030] Dissanayake E. (1992). Homo aestheticus: Where art comes from and why.

[B7-behavsci-15-01030] Galliart J., Frazer W. (2022). Association of American Medical Colleges Group on women in medicine and science, supporting women faculty through art therapy: Participant experiences *[Poster]*. Learn Serve Lead.

[B8-behavsci-15-01030] Hill A. (1948). Art versus illness.

[B9-behavsci-15-01030] Holmes M., Leone L. (2021). Emptying the jar: Crochet to unpack toxic racial stress. Craft in art therapy: Diverse approaches to the transformative power of craft materials and methods.

[B10-behavsci-15-01030] Inman S. L. (2018). Mandala workshop: An affirming mandala workshop for healthcare professionals *[Unpublished Master’s project]*.

[B11-behavsci-15-01030] Isis P. D., Gussak D., Rosal M. (2016). Positive Art Therapy. The Wiley handbook of art therapy.

[B12-behavsci-15-01030] Jalambadani Z., Mahmoudi R., Assarzadeh H., Ghorbani A. R., Shegarf Nakhaie M. (2025). The impact of mindfulness-based art therapy on the quality of life, lifestyle, and health literacy of breast cancer patients. Art Therapy.

[B13-behavsci-15-01030] Jasso P. (2018). Highlights of the KAC/ESU arts in medicine partnership *[Paper presentation]*. National Assembly of State Arts Agencies Bi-Annual Convening in 2018.

[B14-behavsci-15-01030] Kaiser D. H. (2017). Special issue on medical art therapy. Art Therapy.

[B15-behavsci-15-01030] Kansas Arts Commission (2025). The kansas arts commission.

[B16-behavsci-15-01030] Kapitan L., Leone L. (2021). Crafting the artist book as embodied, relational practice. Craft in art therapy: Diverse approaches to the transformative power of craft materials and methods.

[B17-behavsci-15-01030] Lamb N. M. (2018). Shrinky Dinks^®^ and cancer care: A personal symbol workshop with a cancer support group *[Unpublished Master’s project]*.

[B18-behavsci-15-01030] Leone L. (2021). Craft in art therapy: Diverse approaches to the transformative power of craft materials and methods.

[B19-behavsci-15-01030] Mageary J., Leone L. (2021). Zines, the DIY ethic, and empowering marginalized identities. Craft in art therapy: Diverse approaches to the transformative power of craft materials and methods.

[B20-behavsci-15-01030] Malchiodi C. A. (1993). Art and medicine [Special issue]. Art Therapy: Journal of the American Art Therapy Association.

[B21-behavsci-15-01030] Malhotra B. (2022). My journey exploring medical art therapy for burn care: From practice to applied research.

[B22-behavsci-15-01030] Potash J. S. (2018). Special issue on medical art therapy. Art Therapy: Journal of the American Art Therapy Association.

[B23-behavsci-15-01030] Rubin J. A. (2009). An introduction to art therapy: Sources and resources.

[B24-behavsci-15-01030] SAMSHA (2014). SAMHSA’s concept of trauma and guidance for a trauma-informed approach.

[B25-behavsci-15-01030] Seligman M. E. P. (2002). Authentic happiness: Using the new positive psychology to realize your potential for lasting fulfillment.

[B26-behavsci-15-01030] Seligman M. E. P. (2011). Flourish: A visionary new understanding of happiness and well-being.

[B27-behavsci-15-01030] Shonkoff J. P., Garner A. S. (2012). The committee on psychosocial aspects of child and family health, and the committee on early childhood, adoption, and dependent care: The lifelong effects of early childhood adversity and toxic stress. Pediatrics.

[B28-behavsci-15-01030] Thompson E. R. (2007). Development and validation of an internationally reliable short-form of the positive and negative affect schedule (PANAS). Journal of Cross-Cultural Psychology.

[B29-behavsci-15-01030] Wolf Bordonaro G. P. (2023). Reconceptualizing self-care as an ethical mandate: A virtual arts-based positive psychology workshop *[Paper presentation]*. The Kansas Colorectal Cancer Control Program (CRCCP) ECHO Series (Online Education).

[B30-behavsci-15-01030] Wolf Bordonaro G. P., Blake A., Corrington D., Fanders T., Morley L. (2009). Exploring media processes and project applications: (Re)Discovering Shrinky Dinks^®^. *Arts and Activities*.

[B31-behavsci-15-01030] Wolf Bordonaro G. P., Galliart J., Haoyu H., Verburg A., VanSteenhuyse K. (2023). Arts in medicine: An Emporia State University, KAC, KU Medical Center Collaboration. 54th Annual American Art Therapy Association Conference.

[B32-behavsci-15-01030] Wolf Bordonaro G. P., Jasso P., Inman S., Lamb N. (2019a). The Emporia State University and Kansas Creative arts industries commission arts in medicine program: A partnership highlighting art therapy as an arts commerce construct. National Conference of the American Art Therapy Association.

[B33-behavsci-15-01030] Wolf Bordonaro G. P., Jasso P., Weeks Neuburger C. W., Emanuel N., Jacobs S. (2019b). Art therapy as a welcomed guest in Kansas’ fine arts communities. National Conference of the American Art Therapy Association.

[B34-behavsci-15-01030] Woolhiser Stallings J., Clark S., Leone L. (2021). Healing with fire: The use of hot glass in art therapy. Craft in art therapy: Diverse approaches to the transformative power of craft materials and methods.

[B35-behavsci-15-01030] Yi S., Leone L. (2021). Demystifying the individualistic approach to self-care: Sewing as a metaphorical process for documenting relational and communal care in disability culture. Craft in art therapy: Diverse approaches to the transformative power of craft materials and methods.

